# Endogenous Viral Elements in Ixodid Tick Genomes

**DOI:** 10.3390/v15112201

**Published:** 2023-10-31

**Authors:** Miranda Barnes, Dana C. Price

**Affiliations:** Center for Vector Biology, Department of Entomology, Rutgers, The State University of New Jersey, New Brunswick, NJ 08901, USA; mirandabarnes@ufl.edu

**Keywords:** endogenous viral elements, ticks, *Ixodes*, paleovirology, arthropod viruses, *Bunyavirales*, *Mononegavirales*, *Rhabdoviridae*

## Abstract

The documentation of endogenous viral elements (EVEs; virus-derived genetic material integrated into the genome of a nonviral host) has offered insights into how arthropods respond to viral infection via RNA interference pathways. Small non-coding RNAs derived from EVE loci serve to direct RNAi pathways in limiting replication and infection from cognate viruses, thus benefiting the host’s fitness and, potentially, vectorial capacity. Here we use informatic approaches to analyze nine available genome sequences of hard ticks (Acari: Ixodidae; *Rhipicephalus sanguineus*, *R. microplus*, *R. annulatus*, *Ixodes ricinus*, *I. persulcatus*, *I. scapularis*, *Hyalomma asiaticum*, *Haemaphysalis longicornis*, and *Dermacentor silvarum*) to identify endogenous viral elements and to illustrate the shared ancestry of all elements identified. Our results highlight a broad diversity of viral taxa as having given rise to 1234 identified EVEs in ticks, with *Mononegavirales* (specifically *Rhabdoviridae*) well-represented in this subset of hard ticks. Further investigation revealed extensive adintovirus integrations in several *Ixodes* species, the prevalence of *Bunyavirales* EVEs (notably not observed in mosquitoes), and the presence of several elements similar to known emerging human and veterinary pathogens. These results will inform subsequent work on current and past associations with tick species with regard to the viruses from which their “viral fossils” are derived and may serve as a reference for quality control of various tick-omics data that may suffer from misidentification of EVEs as viral genetic material.

## 1. Introduction

In the absence of an adaptive immune system, RNA-mediated silencing pathways such as RNAi constitute a valuable tool through which many arthropods can protect themselves from the adverse consequences of viral infection [1]. These pathways utilize the RNA template to recognize and repress infection by base-pairing and cleaving RNAs derived from cognate virus. One such source of templates are transcription products of endogenous viral elements (EVEs), or segments of genetic material from infecting viruses that have become integrated into the genome of a host (nonviral) organism and are passed down to successive generations [2]. Though many EVE origins can be traced to retroviruses that insert DNA-based copies of their RNA genomes into host cellular DNA (and may undergo mutation-induced loss of function over time) [3,4], RNA viruses that do not possess the reverse transcriptase enzymes necessary to convert their genetic material into cDNA can still become nonretroviral integrated RNA viral sequences (NIRVS; a class of EVE) if they co-opt such enzyme activity from elsewhere, particularly from the long terminal repeat (LTR) retroelements around which NIRVS are often clustered [5]. The exact reasons as to why certain nonretroviruses (particularly negative-sense, single-stranded RNA viruses) appear to be integrated more often than others remain unclear, but duration of infection (acute versus persistent), length of RNA sequence, and replication location (in the cytoplasm—as is typical for most RNA viruses—as opposed to within the nucleus) have all been suggested as potential factors influencing reverse transcription and integration frequency [6].

Of the several different types of RNAi described (i.e., microRNA, small interfering RNA, etc.), the PIWI-interacting RNA pathway (piRNA) represents a primary means through which EVEs are mobilized for antiviral function in some arthropods, as evidenced by a high concentration of EVEs in piRNA clusters coupled with their low prevalence among small interfering RNAs (siRNAs; otherwise considered one of the central systems through which arthropods defend themselves against invading viruses) in these organisms [7]. Though PIWI-interacting RNAs have been traditionally associated with the suppression of transposable elements (TEs) in both somatic and germline cells [8,9], the use of this mechanism for the targeting and degradation of viral RNA has been increasingly well-documented, particularly in mosquitoes [10,11]. Several studies have confirmed the functioning of piRNA pathways in response to viral infection in the lab [12,13,14,15], and EVE-derived piRNAs have been shown to play a direct role in curtailing the replication of the cell-fusing agent virus in the ovaries of *Aedes aegypti* [16]. As one of many potential internal factors influencing a tick’s ability to contract, survive, and maintain an infection in a way that facilitates passage to a vertebrate host, further characterizing piRNAs and the EVEs from which they are derived is necessary to begin to understand the interplay between pathogen virulence, host clearance, and tolerance as an immune strategy and may ultimately have implications for the comprehension and control of arbovirus transmission.

The presence of an EVE in a genome does not necessarily imply a functional role in tick viral regulation, and even once-useful EVEs are likely to become degraded through the accumulation of mutations over time. Even so, documenting EVEs can provide valuable information to researchers about the histories of viral exposure and/or infection within a given species or lineage. After genomic integration, various evolutionary pressures will determine whether an EVE is retained or lost: for example, a deleterious integration into a functional gene is unlikely to become established in a population, but EVE introduction to a site that poses a negligible detriment to the affected individuals (or even offers a slight advantage) may be maintained across multiple generations [17]. Sequences integrated into the ancestors of various hosts represent a snapshot of a point in viral evolution that would be difficult to reconstruct from extant viral lineages [2,17,18]. This principle underlies direct paleovirology, which has allowed researchers to gauge the approximate timescale on which ancient infections occurred relative to species divergence, reconstruct and analyze long-extinct viruses to assess how host–virus interaction has changed over time, and develop new insights into EVEs that have undergone exaptation and now serve a useful purpose in their hosts [18], as is the case with EVEs in the piRNA pathway.

Considering their significance to both vector immunology and paleovirology, our analysis aims to contribute to the current discussion on arthropod EVEs by identifying and reporting EVE loci in the genomes of nine ticks (*Dermacentor silvarum, Hyalomma asiaticum, Haemaphysalis longicornis, Ixodes persulcatus, I. ricinus, I. scapularis, Rhipicephalus annulatus, R. microplus,* and *R. sanguineus*), comparing the sequences’ prevalence, phylogeny, and relation to disease in humans and livestock. Six of these genomes are newly sequenced and high-quality contiguous assemblies, with many anchored to chromosomes [19], affording new opportunities to screen for such elements with recent data.

## 2. Materials and Methods

Assembled tick genomes were downloaded from the National Center for Biotechnology Information databases; when applicable, both RefSeq and GenBank assemblies were used (i.e., *Dermacetor silvarum*, *Ixodes scapularis*, and *Rhipicephalus sanguineus*; Table 1), as we have noted that EVE-encoding scaffolds may be removed from RefSeq during contaminant screening. The tick species in this analysis were selected primarily based on the availability of fully sequenced, assembled, and publicly available genomes, though all are relevant vectors of human disease [20,21,22,23,24] and/or significant ectoparasites on livestock [25,26] in different locations around the world.

Putative EVE loci were identified as follows: first, all viral proteins were retrieved from the National Center for Biotechnology Information (NCBI) viral protein database (35.3 M sequences as of 30 May 30 2021) [30] and clustered using cd-hit [31] at a 95% amino acid identity level, resulting in 10.66 M representative sequences that were used in a TBLASTN query against the tick genome references (*e*-val = 1 × 10^−5^). The BLAST output was parsed, and the reference nucleotide coordinates were used to extract the aligned portion of the genomic sequence. We noted in many cases that multiple overlapping BLAST alignments were reported for the same genome locus due to features such as mutation-derived frameshifts; we thus used CD-HIT-EST [31] to cluster the extracted nucleotide sequences at 100% sequence identity. As we did not cluster sequences that are not completely overlapping, we manually examined the DIAMOND output and derived the left- and rightmost coordinates for the putative EVE loci with offset yet overlapping local alignments and top hits to the same virus. Second, to help ensure the viral provenance of the locus, we added 200 bp of upstream and downstream genomic nucleotide sequence to the extracted genome subsequence and performed another protein homology search by using DIAMOND (BLASTX algorithm) in ultra-sensitive mode, using this new query against the entire NCBI ‘nr’ protein database (retrieved July 2021; *e*-val = 1 × 10^−5^) with the top ten high-scoring pairs (HSPs) saved. The results were divided into two categories: (1) queries (loci) with top-scoring hits to viruses, and (2) queries with a top hit to a eukaryote (generally other ticks) that included a viral hit among the top ten HSPs. This was necessary to identify EVE-derived ORFs in public tick genomes that carry eukaryote annotations due to automated pipelines and would otherwise lack identification by focusing strictly on top hits that were assigned a viral taxonomy. DIAMOND hits were assigned an NCBI taxonomic string by using the taxonomizr R module (https://cran.r-project.org/web/packages/taxonomizr/ accessed on 14 July 2022), and the associated nucleotide CDS (or coding) sequence was extracted for each, using the NCBI command-line e-utilities.

There has yet to be a uniform methodology established for ensuring consistency in EVE results, both in regard to the steps taken to identify EVEs and the way individual EVEs identified as multiple BLAST HSPs should be classified (i.e., combined into one EVE or as several secondary EVEs), though more standardized approaches have recently been proposed [32]. In this analysis, discrete hits were treated as single EVEs where clear overlap occurred. To eliminate redundancy in our output, including duplicate versions of the same EVEs identified in RefSeq and GenBank assemblies for *Dermacentor silvarum*, *Ixodes scapularis*, and *Rhipicephalus microplus*, cd-hit-est was used to cluster nucleotide sequences for all potential EVEs, as specified above. The amino acid translations corresponding to the identified tick EVE loci and their NCBI top viral hits were extracted from the DIAMOND output and aligned using the MAFFT E-INS-i algorithm [33]. The resulting un-partitioned alignment was used to create a consensus maximum-likelihood phylogenetic tree, using IQ-TREE with automatic model selection [34] and branch supports determined by 2000 rapid bootstrap replicates. We elected to construct separate phylogenetic analyses (exclusive of the putative remaining NIRV-like EVEs; discussed below) for the identified adintoviral and iridoviral elements due to their large number, conservation, and potential status as replicative DNA elements.

## 3. Results

After clustering HSPs at 100% nucleotide identity, +/− 200 bp extension, and DIAMOND BLASTX search against the full NCBI ‘nr’ database, 1234 genomic loci were retained for further analysis (full DIAMOND homology output available in Supplementary Table S1) after manually clustering overlapping HSPs. Together, these loci had top hits spanning 208 unique reference amino acid accessions in NCBI, indicating a high degree of redundancy and multiplicity in EVE integration referencing currently described viruses. Of the 742 EVE loci identified with top BLAST hits to eukaryote proteins, 638 (85.8%) were annotated as hypothetical, putative, or uncharacterized proteins, while 90 (12.1%) were annotated as a reverse transcriptase; 325 (43.7%) had a top viral HSP to *Adintoviridae*, with an additional 121 (16.2%) and 111 (14.9%) to *Iridoviridae* and *Parvoviridae* respectively. The mean length of the HSP alignment for this set of hits was 222 bp, whereas that for the respective viral top hit for each was 230 bp, indicating that the alignments for these putative EVE loci to viral references were not significantly shorter than that of the eukaryote reference despite the latter exhibiting a higher BLAST bitscore.

**Figure 1:**
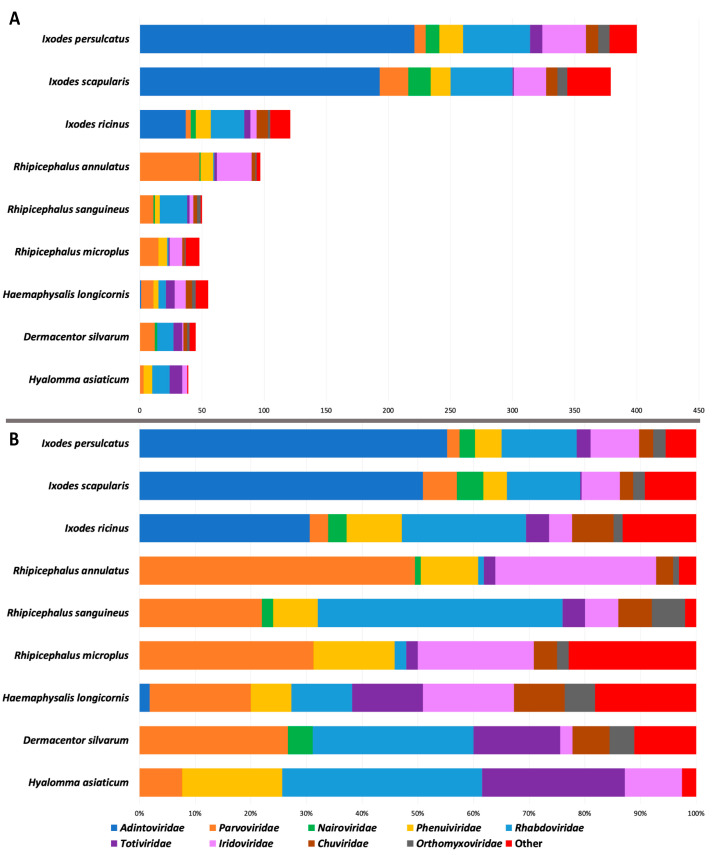
Distribution and abundance of tick endogenous viral elements identified in this study. Viral families with >10 total elements are represented, while those with fewer (*Baculoviridae*, *Flaviviridae*, *Partitiviridae*, *Polydnaviridae*, *Poxviridae*, *Siphoviridae*, and unclassified viruses) are grouped within “Other”. The total number of identified elements (**A**) and relative proportion (**B**) are illustrated on the *x*-axis.

Thirteen viral orders comprising 15 families were represented in the output (Supplementary Tables S1 and S2; Figure 1): *Mononegavirales* (*Rhabdoviridae*), *Jingchuvirales* (*Chuviridae*), *Bunyavirales* (*Nairoviridae*, *Phenuiviridae*), and *Articulavirales* (*Orthomyxoviridae*) are negative-sense single-stranded RNA viruses; *Amarillovirales* (*Flaviviridae*) is a positive-sense single-stranded RNA virus; *Durnavirales* (*Partitiviridae*) and *Ghabrivirales* (*Totiviridae*) are double-stranded RNA viruses; *Piccovirales* (*Parvoviridae*) is a single-stranded DNA virus; and *Siphoviridae* (*Caudovirales*), *Lefavirales* (*Baculoviridae*), *Orthopolintovirales* (*Adintoviridae*), and *Pimascovirales* (*Iridoviridae*) are dsDNA viruses. The *Orthopolintovirales* was the most abundant viral order recovered, with 453 identified EVE integrations, all of which were derived from adintovirus integrase sequences and found in ticks of the genus *Ixodes*, primarily *I. persulcatus* and *I. scapularis* (Figure 1 and Supplementary Figure S2). Similar to the *Adintovirales*, a large number of elements with homology to *Pimascovirales* (*n* = 121) were identified. These largely comprised top hits to reverse transcriptase enzymes of Erythrocytic necrosis virus (*n* = 78) and Lymphocystis disease virus 4 (*n* = 42), indicating that these elements are derived from invertebrate-infective *Iridoviridae* ([35]; Supplementary Figure S3), a lineage of nucleocytoplasmic large DNA viruses that, although not known to exhibit canonical transposon-like behavior, can encode eukaryotic class II DNA transposons [36]. Because these EVEs may be governed by mechanisms more closely related to the replication of transposable DNA elements or retroviruses than the dynamics shaping horizontal transmission and the integration of exogenous NIRV precursors, and to simplify further computational steps and better highlight the relationships between the remaining viral hits, these adintoviral and iridoviral sequences were removed from the inclusive phylogenetic analysis and used to construct separate phylogenies so as to discern their pre- or post-speciation proliferation.

*Mononegavirales* was the most abundant non-adintoviral or iridoviral order identified, comprising 208 total identified EVEs present in all nine genomes analyzed. A majority of these were from the family *Rhabdoviridae* (*n* = 116; 55.8%); however, as Norway mononegavirus (currently unplaced) has been recovered within the *Rhabdoviridae* in previous phylogenetic analyses [37], this number rises to 186 or 89.4%. This is consistent with EVE identifications performed thus far in mosquitoes, which are also biased toward various members of *Rhabdoviridae* [7]. *Piccovirales* represented the second most abundant order (*n* = 135); all strains were members of *Parvoviridae* and largely clustered into three major clades (VP1, NS1, and ORF1; discussion below). The third most abundant order, *Bunyavirales* (116 identified EVE integrations), is of interest due to its relative prevalence in several of these tick genomes. Though *Bunyavirales*-derived EVEs do appear in a multitude of arthropod genomes, they are often outnumbered by other viral orders [7,32]; in this analysis, they were identified in all nine tick genomes and were abundant in multiple species. Due to the fact that viral taxonomy within the order *Bunyavirales* is currently in flux (several of the viral top hits reported were only granted generic rank the prior year [38]), there may be additional EVEs derived from this lineage (and other viral orders) with even greater homology to loci identified herein that await discovery and/or classification.

**Figure 2:**
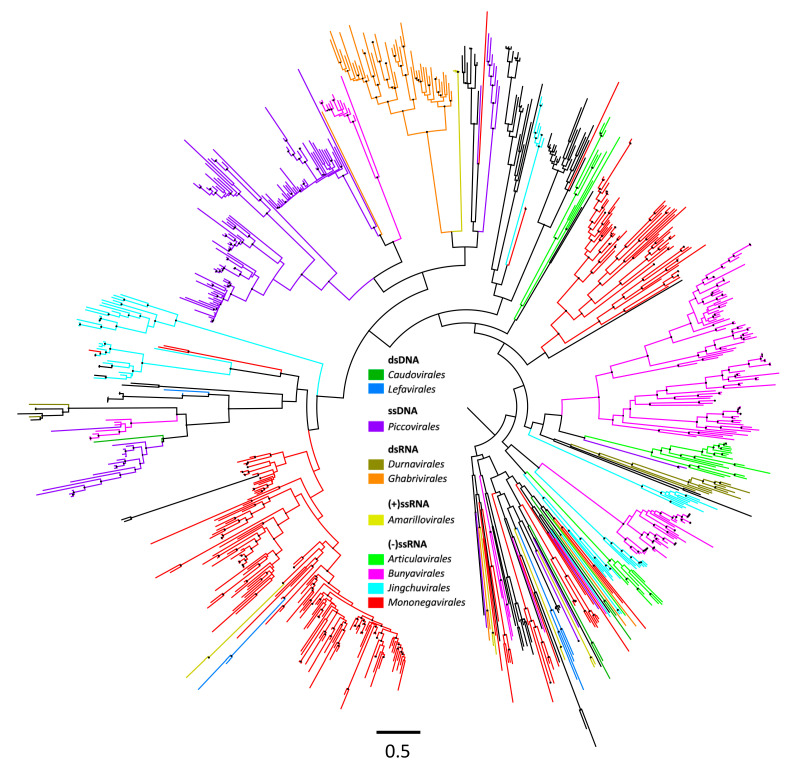
Maximum-likelihood radial phylogeny of EVE elements identified in hard tick genomes. The tree is manually rooted on *Polydnaviridae*. Lineages are color-coded according to viral order (inset); nodes with bootstrap supports ≥90 are represented with black circles. The fully annotated tree is available in Supplementary Figure S1.

The tick genomes analyzed here varied broadly both in terms of number of EVE integrations and in the predominant viral lineages that comprised them (Table S2; Figure 2). *Mononegavirales* had the greatest number of EVE loci in all but *H. longicornis, R. annulatus*, and *R. microplus*, excluding adintoviral and iridoviral sequences (which were most prevalent in *I. persulcatus*); in each of these three genomes, the most abundant elements were derived from *Parvoviridae*, perhaps reflecting a disparity in the past host associations of these ticks over the course of their evolutionary trajectories. The *Ixodes* ticks encoded the largest number of EVEs overall (*n* = 121 in *I. ricinus*; *n* = 400 in *I. persulcatus*), even after accounting for the latter retroelements. By contrast, the remaining tick genomes encoded between 39 and 97 EVEs. This disparity between tick genomes in EVE numbers and diversity is of particular interest; the two species with the fewest *Mononegavirales*-derived EVEs (*Rhipicephalus annulatus* and *Rhipicephalus microplus*, each with only a single rhabdoviral EVE identified) retained abundant parvoviral EVEs yet are both single-host ticks (all three life stages feed and develop on the same host [39,40]) and thus may have fewer opportunities to acquire viruses from multiple diverse vertebrates than do the other ticks featured in our analysis which utilize three hosts during their life cycle [21,41,42,43,44,45,46].

Many of the NCBI top hits to viral EVEs identified herein remain largely uncharacterized beyond their detection in broad analyses of arthropod viromes; it is thus difficult to infer the significance of these elements to the tick’s paleovirological history and whether they reflect common environmental challenges or perhaps even pathogenic arboviruses. As selective pressures on these genetic elements are relaxed after incorporation into the host genome, most EVEs have undergone extensive sequence degeneration via neutral mutation over many millions of years; thus, homology to extant viruses remains very low. The average DIAMOND amino acid identity among all 782 non-adintoviral EVEs reported here was 48.14%, with only 10 such elements (1.3%) retaining >90% identity and 71 elements (9.0%) retaining >70%.

## 4. Discussion

**Figure 3:**
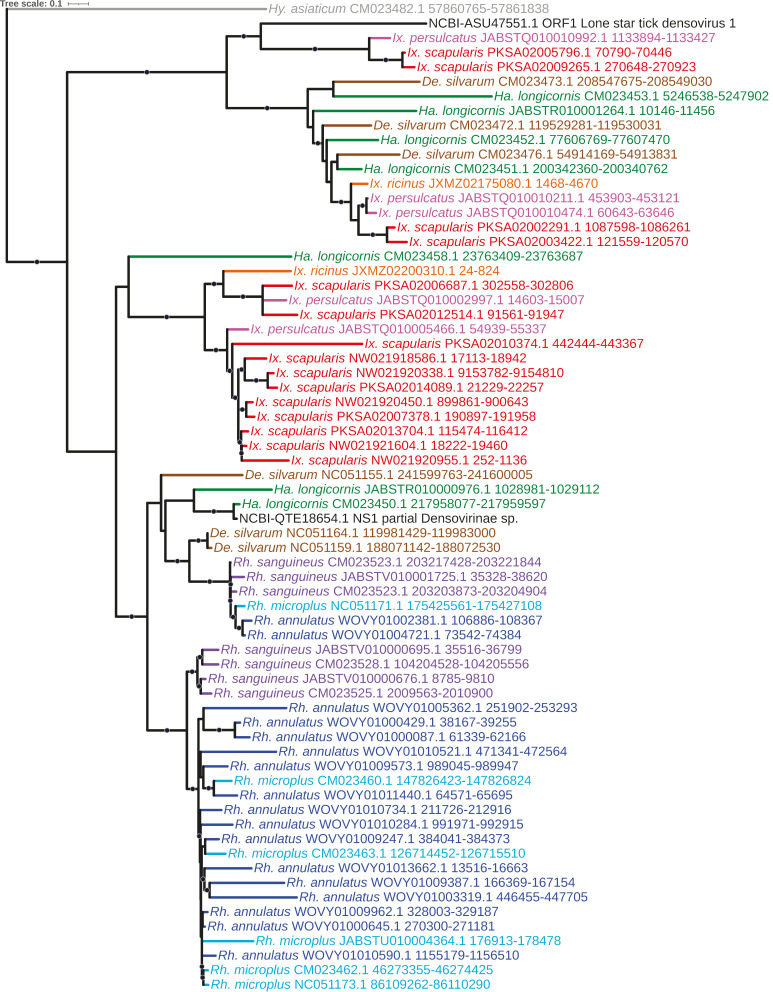
Sub-clade of phylogeny generated in Figure 2 illustrating shared ancestry of parvoviral EVEs identified in tick genomes derived from NS1-encoding elements. Nodes with bootstrap supports ≥90 are represented with black circles. Tip labels for EVEs are of the format “(tick species) (scaffold accession) (coordinates)”; labels for the NCBI top hits are of the format “NCBI—(accession) (annotation) (virus)”. Taxa are color-coded according to tick species (inset).

The phylogeny constructed from these identified EVE loci and NCBI reference hits (Figure 3; the fully annotated tree is available as Supplementary Figure S1; the adintoviral and iridoviral phylogenies are available as Supplementary Figures S2 and S3, respectively) contained a disparate mix of genes and gene fragments from both closely and distantly related viruses; as such, it was not interpreted as illustrative of interrelationships of all constituent EVEs but rather to highlight strongly supported (bootstrap support ≥90) clades that comprise unique lineages of these genomic elements. Using this phylogenetic tree, we identified several major lineages of endogenized viruses for further discussion.

### 4.1. Double-Stranded DNA (dsDNA) Viruses

We identified 453 EVE integrations with homology to *Adintoviridae* (order *Orthopolintovirales*). Adintoviruses were, until recently, believed to be transposable elements known as polintrons or Mavericks, but evidence of the existence of corresponding viral capsid proteins in these sequences has resulted in their reclassification as double-stranded DNA viruses that integrate into host genomes as part of their replicative cycle [47]. Notably, this suggests that, unlike many of the other viral hits (which do not possess their own integrases or among the RNA viruses, reverse transcriptases), the adintoviruses found in these tick genomes may have facilitated the process of incorporation themselves. Such elements are widespread in eukaryotic genomes, and although no comparable analysis across arthropods has been conducted, phylogenetic studies in vertebrates suggest both horizontal and vertical transmission and document both organisms in which these sequences appear with remarkably high frequency (albeit often in degraded forms), as well as lineages in which they have been lost entirely [48]. The former event (not unlike transposon proliferation) may have occurred in a common ancestor of the *Ixodes* in our analysis, with the exact copy number for each species diverging with evolutionary time. The phylogeny generated from these elements (Supplementary Figure S2) illustrates a high degree of within-genome duplication in both *Ix. scapularis* and *Ix. persulcatus* that is absent from *Ix. ricinus*, suggesting that the spread of these elements was silenced during the speciation of the latter.

The iridoviral elements (Supplementary Figure S3) exhibit enigmatic low-scoring top hits to known infective viruses of fish. To our knowledge, iridoviruses have not been identified in ticks; however, the causative agent of African swine fever virus (which has been detected in soft and hard ticks) is hypothesized to represent a sole lineage of ancestral nucleocytoplasmic large DNA viruses (NSLVs) that eventually diversified into known *Megaviricetes*, including *Pimascovirales* [49]. The EVEs described herein may thus be artifacts of an ancient association between ticks and this putative virus, as the resultant phylogeny assorts predominantly into alternating clades consisting of *Ixodes* species or of *Rhipicephalus*, *Haemaphysalis*, and *Hyalomma*. The NCBI reference sequences cluster to the exclusion of all other EVEs in this clade, likely driven by the fact that extant viruses from which the references were generated over the past decade remain more conserved at the sequence level than neutrally evolving EVEs over millennia, as well as by long-branch attraction artifacts that associate the full-length RdRp references exclusive of the shorter EVE fragments.

Additional *Polydnaviridae*-derived elements (unassigned to taxonomic order) of the genera *Ichnoviridae* and *Bracoviridae* were detected in the genomes of the *Ixodes* species analyzed. A *Cotesia congregata* bracovirus-like (CcBV) EVE was identified in the *Ixodes scapularis* genome; this and other members of *Polydnaviridae* are best-known for their symbiotic association with parasitoid wasps. When a wasp deposits its eggs, the virus is able to infect the cells of the parasitoid’s host, thus weakening it and facilitating the survival of the developing larvae [50]. As double-stranded DNA viruses encoding their own integrases, they are capable of inserting themselves into host genomes, allowing wasps to pass them from parent to offspring as integrated proviruses; however, because all wasp somatic and germline cells already possess integrated virus, the integrase is primarily used to remove rather than insert sequences during assembly of the viral particles that will be introduced to the immature wasp’s host organism [51]. The homology between this *I. scapularis* element was strongest to the CcBV protein 31.2 (Supplementary Table S1), encoding a retroviral integrase that itself has been hypothesized to be of exogenous origin (or integration of another smaller viral integrase) to CcBV due to its identification in the nematodes *Caenorhabditis* elegans and *C. briggsae* and the beetle *Tribolium castaneum* [52]. Phylogenetically, the protein sequence of the *I. scapularis* element and the NCBI reference top hit (YP_184882.1; *Cotesia congregata* bracovirus) cluster with an *I. scapularis*-derived MELD (midsize eukaryotic linear dsDNA) virus and its associated reference (DAC81743.1; *Trichoplax* MELD virus) with a bootstrap support of 100%. MELD viruses are a lineage of Adintovirus, but unlike the rest of the *Adintoviridae*, it was not removed from the phylogenetic analysis due to its unassigned NCBI taxonomy. These data indicate that the bracovirus-like EVEs may not be derived from the *Bracoviridae* but rather from the ancestral virus that itself is represented as the CcBV 31.2 element. In addition to bracoviruses, a clade comprising *I. scapularis* and *I. persulcatus* elements with homology to *Apophua simplicipes* ichnovirus was identified in the analysis and clustered with the two NCBI reference hits (*A. simpliceps* ichnovirus and *Glypta fumiferanae* ichnovirus) with a bootstrap support of 100%.

Given that no members of the *Braconidae* or *Ichneumonidae* (the traditional partners for polydnavirus symbioses [50]) were known to parasitize tick hosts until very recently (*Ixodiphagus* belong to the *Encyrtidae*, and the single putative braconid parasitoid recorded has yet to be fully characterized) and integrase genes are not incorporated into viral particles produced by the wasp, opportunities for polydnavirus endogenization are likely to be rare [51]. It is thus likely that these elements do not represent genuine polydnavirus integrations and are instead retroviral genes.

### 4.2. Single-Stranded DNA (ssDNA) Viruses

**Figure 4:**
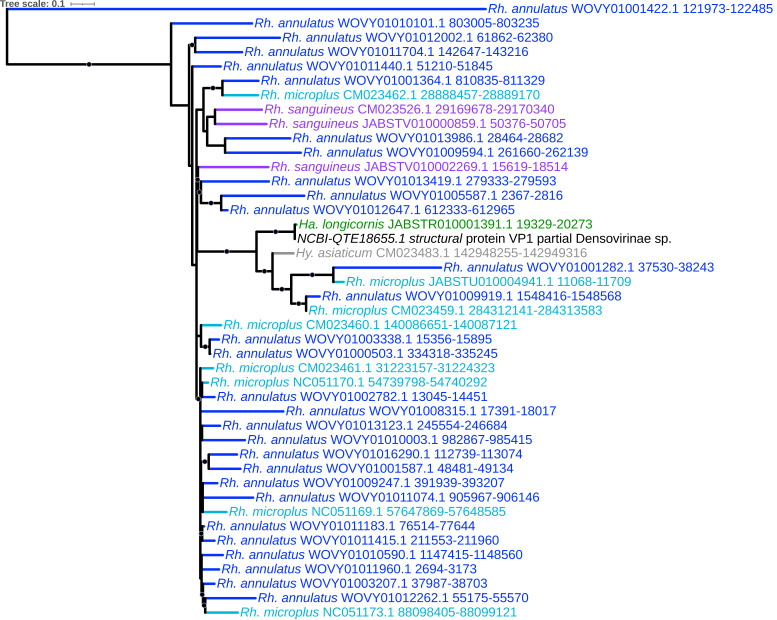
Sub-clade of phylogeny generated in Figure 2 illustrating shared ancestry of parvoviral EVEs identified in tick genomes derived from VP1-encoding genes. Nodes with bootstrap supports ≥90 are represented with black circles. Taxon labels are formatted and color-coded by tick species as shown in Figure 3.

We identified a large number (*n* = 135) of EVE elements with homology to *Parvoviridae*. Parvoviruses are small non-enveloped single-stranded ssDNA viruses that are reported to infect both vertebrates and invertebrates, including ticks [53], with vertebrate-infective parvoviruses themselves isolated from ticks [54]. Of these 135 elements, 98 (72.6%) had top viral hits to the nonstructural (NS1) and structural protein VP1 of a *Densovirinae* sp. virus recovered from *H. longicornis* ticks in Liaoning, China [53], and ORF1 of Lonestar tick densovirus 1 (Supplementary Table S1). The NS1-like elements (Figure 3) were identified in all nine tick genomes and formed a monophyletic lineage that further assorted into strongly supported genus- and species-specific subclades containing multiple elements primarily from *Rhipicephalus* and *Ixodes* species, indicating lineage-specific duplication and proliferation of these elements. The VP1-like elements (Figure 4) additionally shared a common ancestor and were present with multiple copies identified in all three *Rhipicephalus* species, while a single element was identified in both *H. asiaticum* and *H. longicornis* (Supplementary Table S1). One pair of NS1 and VP1-like EVEs identified in *H. longicornis* exhibited much greater sequence homology along the length of the HSP (96.7% and 98.9%, respectively) than the remaining parvoviral EVEs in other ticks, suggesting that these elements may have been integrated more recently, perhaps from a lineage closely related to that of the NCBI reference isolated from field-caught *H. longicornis.* A separate monophyletic and highly supported clade of parvoviral-derived EVEs with top hits to ORF1 of Lonestar tick densovirus, isolated from the Lonestar tick *Amblyomma americanum* in the United States [55], was identified with elements in all three *Ixodes* species, *D. silvarum*, *R. sanguineus*, and *H. longicornis*.

### 4.3. Mononegavirales (-ssRNA)

**Figure 5:**
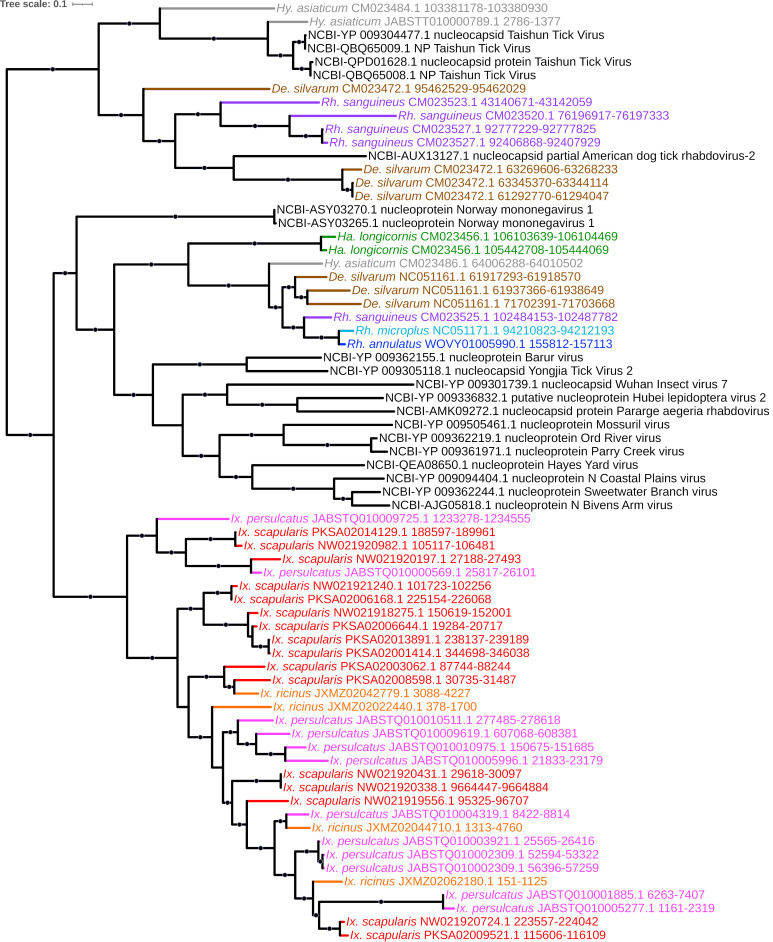
Sub-clade of phylogeny generated in Figure 2 illustrating shared ancestry of mononegaviral EVEs identified in tick genomes derived from nucleoprotein-encoding *Rhabdovirus* elements. Nodes with bootstrap supports ≥90 are represented with black circles. Taxon labels are formatted and color-coded by tick species as shown in Figure 3.

**Figure 6:**
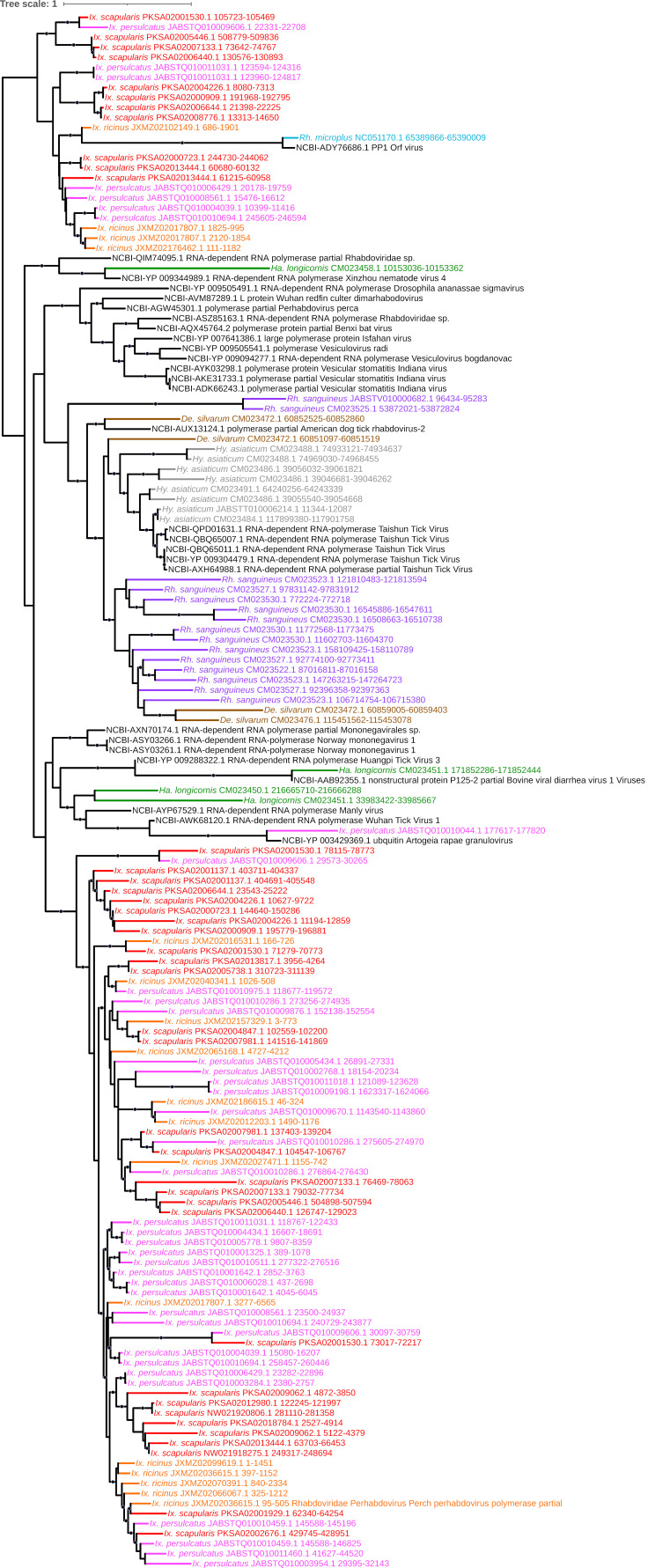
Sub-clade of phylogeny generated in Figure 2 illustrating shared ancestry of mononegaviral EVEs identified in tick genomes derived from RdRp-encoding genes of Norway mononegavirus-like and Taishun tick virus-like ancestry. Nodes with bootstrap supports ≥90 are represented with black circles. Taxon labels are formatted and color-coded by tick species as shown in Figure 3.

The most abundant viral order recovered in our data, the *Monogenavirales*, are a large group of nonsegmented, enveloped viruses currently classified into 11 families and infecting a broad diversity of plants, animals, humans, and frequently reported from metatranscriptomic tick surveys [37,56,57]. All nine tick genomes analyzed here contained EVEs of mononegavirus provenance (*n* = 208), with a majority from the family *Rhabdoviridae* (*n* = 186; 89.4%). This is consistent with EVE analyses of mosquitoes, which are also enriched in various *Rhabdoviridae* [7]. Most of these elements cluster into two major lineages: a clade of nucleoprotein-encoding EVEs with top hits to Rhabdovirus (Figure 5) and a large clade of RNA polymerase (RdRp) elements (Figure 6) that itself comprises strongly supported and monophyletic groups of Norway mononegavirus-like EVEs present only in the three *Ixodes* species; and Taishun tick virus-like elements present in *R. sanguineus*, *D. silvarum*, and *H. asiaticum.* In addition, we identified multiple EVEs in all three *Ixodes* species with homology to Manly and Messner viruses, both with phylogenetic affinity to rhabdoviruses and identified within shotgun metagenomic analyses of reptile-feeding *Amblyomma moreliae* ticks in Australia [58] and *Ixodes uriae* ticks parasitizing Antarctic penguins [59], respectively; this would presumably highlight an ancient association between these viruses and the diverse tick genera that persists today. As metagenomic analyses continue to evolve and pathogen surveillance, identification, and isolation protocols become robust and precise, further characterization of extant viruses that are ecologically or clinically important is likely to occur.

### 4.4. Bunyavirales (-ssRNA)

**Figure 7:**
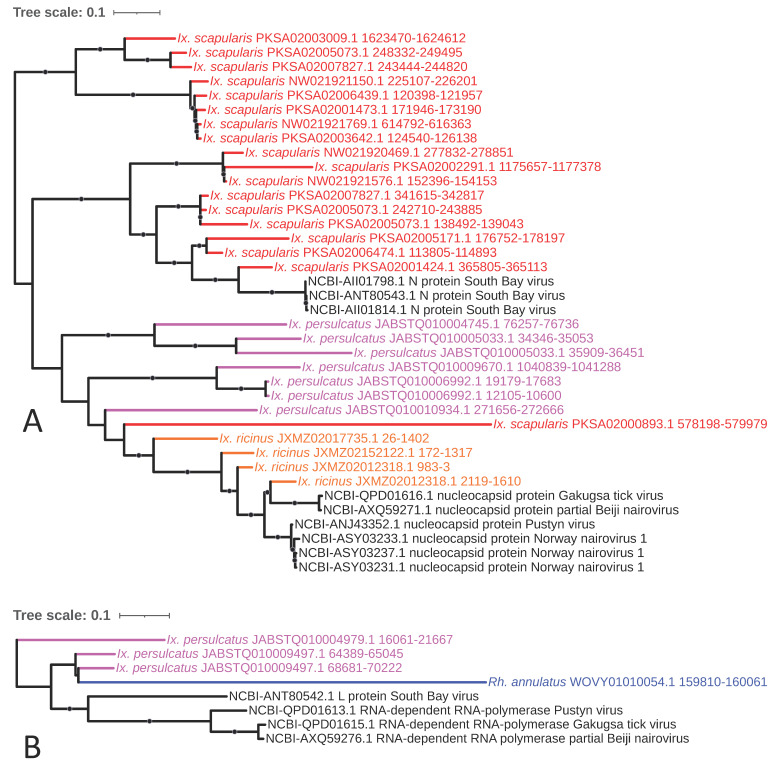
Sub-clades of phylogeny generated in Figure 2 illustrating shared ancestry of bunyaviral EVEs identified in tick genomes. The *Nairoviridae*-like elements comprise independent clades of nucleocapsid-encoding EVEs with homology to the genera *Orthonairovirus* and *Sabavirus* (**A**) and a clade consisting of *Orthonairovirus* RNA polymerase elements (**B**). Nodes with bootstrap supports ≥90 are represented with black circles. Taxon labels are formatted and color-coded by tick species as shown in Figure 3.

**Figure 8:**
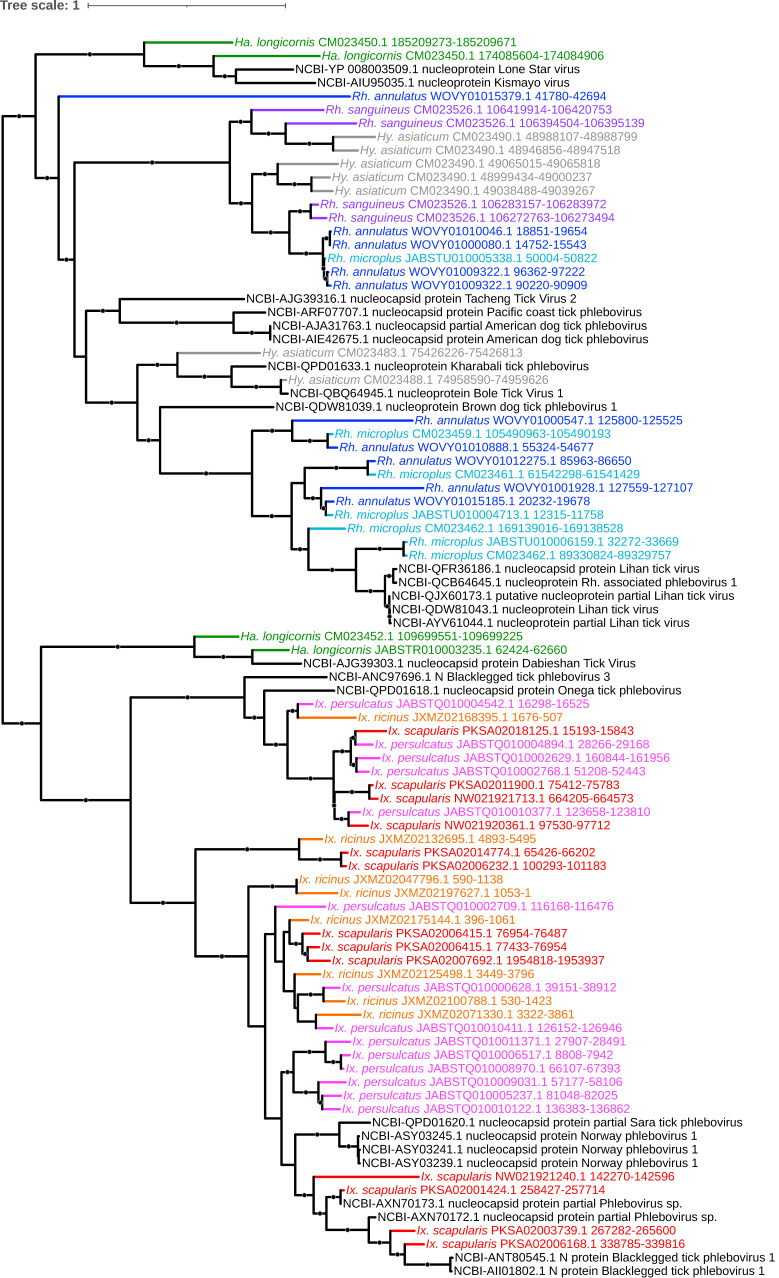
Sub-clade of phylogeny generated in Figure 2 illustrating shared ancestry of bunyaviral EVEs identified in tick genomes. The *Phenuiviridae*-like elements comprise nucleocapsid-encoding EVEs that assort into two major lineages: one of *Ixovirus*- and *Phlebovirus*-like elements present in *Rhipicephalus* species and another comprising *Uukuvirus*- and *Phlebovirus*-like elements in *Ixodes* species. Nodes with bootstrap supports ≥90 are represented with black circles. Taxon labels are formatted and color-coded by tick species as shown in Figure 3.

**Figure 9:**
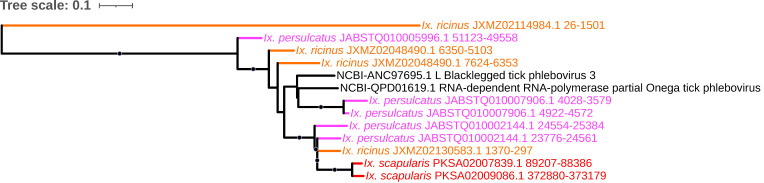
Sub-clades of phylogeny generated in Figure 2 illustrating shared ancestry of bunyaviral EVEs identified in tick genomes. The *Phenuiviridae*-like elements comprise a clade containing RNA polymerase-encoding elements of mixed homology to *Ixovirus* and *Phlebovirus*. Nodes with bootstrap supports ≥90 are represented with black circles. Taxon labels are formatted and color-coded by tick species as shown in Figure 3.

*Bunyavirales* is currently the largest order of RNA viruses, and transmission of bunyaviruses is predominantly achieved via arthropods [60]. The order contains multiple tick-borne human pathogens, such as Severe Fever with Thrombocytopenia Syndrome virus (SFTSV; now Dabie bandavirus), Heartland virus (HRTV), and Bhanja virus (BHAV), among many others. Commensurate with its broad association with ticks, and potentially due to reported germline infection by some members [61] that may potentiate horizontal transmission post-endogenization, we identified a total of 116 EVEs with top hits to *Bunyavirales* spanning all nine genomes analyzed. These elements accounted for 4.6% to 21.3% of total EVEs (excluding *Adintoviridae* and *Iridoviridae*) in each species and accounted for >20% of total EVEs in all three *Ixodes* species, as also reported by Russo et al. in a thorough analysis of an alternative *I. scapularis* assembly [62]. (The assembly chosen here was selected because it comprises fewer contigs and is larger in size and thus is likely to be more complete.) The abundance of such elements within tick genomes is notably higher than in other currently sequenced insect species and vector arthropods, where they represent a comparably small proportion of EVEs in, for example, the mosquitoes *Aedes aegypti* and *Aedes albopictus* (1.9% and 1.3%, respectively [63]). Four major lineages of *Bunyavirales*-derived EVEs that span both the families *Phenuiviridae* and *Nairoviridae* are highlighted: The *Nairoviridae*-like elements comprise independent clades of (1) nucleocapsid-encoding EVEs confined to the *Ixodes* species that assort based on existing homology to the genera *Orthonairovirus* and *Sabavirus* containing only the *Ixodes* species (Figure 7A) and (2) a clade consisting of three *Orthonairovirus* RNA polymerase elements detected in *I. persulcatus* only (Figure 7B). The *Phenuiviridae*-like elements comprise (3) nucleocapsid-encoding EVEs that assort into independent lineages, one comprising *Ixovirus*- and *Phlebovirus*-like elements found only in the three *Ixodes* species and another comprising *Uukuvirus*- and *Phlebovirus*-like elements within *Rhipicephalus* and *Hyalomma* species (Figure 8), and (4) a clade containing RNA polymerase-encoding elements of mixed homology to *Ixovirus* and *Phlebovirus* within all *Ixodes* species (Figure 9) possibly representing EVE integration from the same virus that gave rise to the ixoviral/phleboviral nucleocapsid elements in number three above, as the homology to Onega tick phlebovirus persists in both. 

### 4.5. Jingchuvirales (-ssRNA)

**Figure 10:**
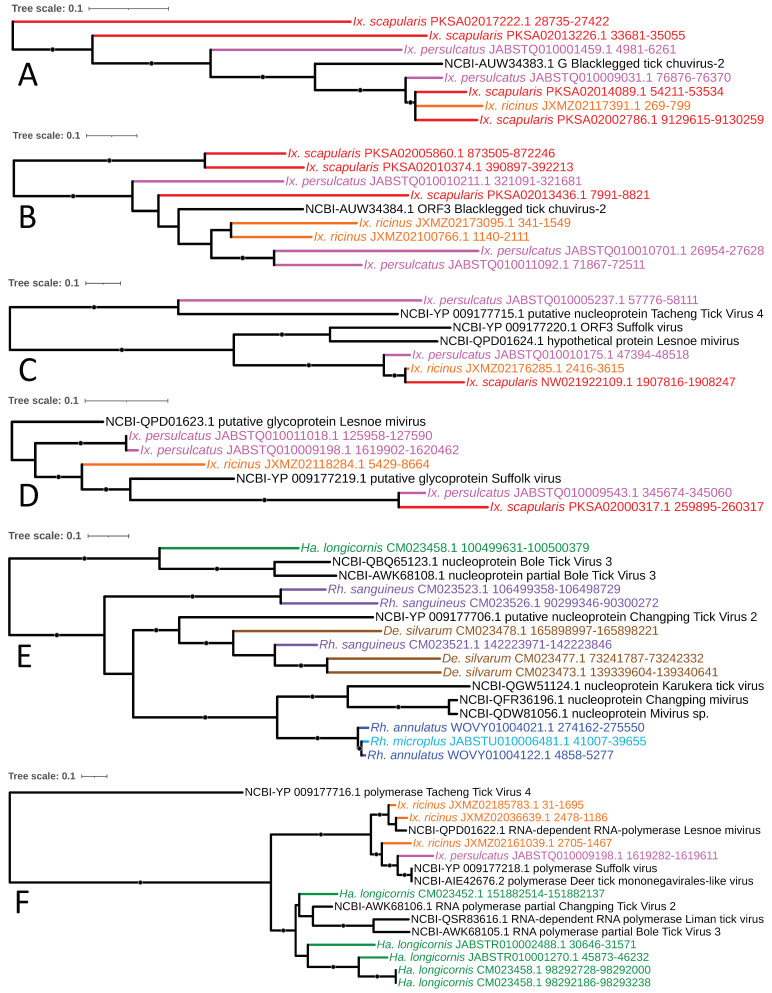
Chuviral EVE elements identified in tick genomes. Sub-clades of phylogeny generated in Figure 2 illustrating shared ancestry of *Chuviridae* EVEs identified in tick genomes comprising a glycoprotein-encoding element with homology to Blacklegged tick chuvirus-2 (genus *Nigecruvirus*; (**A**); a lineage of elements with top hits to ORF3 (otherwise reported as a nucleoprotein, N gene) of the previous *Nigecruvirus* (**B**); a lineage comprising ORF3/hypothetical protein-encoding elements with top hits to members of genus *Mivirus* (**C**); a lineage comprising ORF3/hypothetical protein-encoding elements with top hits to members of genus *Mivirus* (**D**); a nucleoprotein-encoding element with top hits to various *Mivirus* strains present in all three *Rhipicephalus* species examined, *De. silvarum*, and *Ha. longicornis* (**E**); and an RNA polymerase-encoding element with top hits to various *Mivirus* strains detected in *Ha. longicornis* and *Ix. ricinus* (**F**). Nodes with bootstrap supports ≥90 are represented with black circles. Taxon labels are formatted and color-coded by tick species as shown in Figure 3.

The order *Jingchuvirales* was characterized in 2015 and elevated in 2018 as a sister order to *Mononegavirales* that contains viruses (most of which are placed in the family *Chuviridae*) broadly dispersed throughout multiple arthropod orders [64,65]. (Chuviral-like endogenous viral elements have been identified throughout an equally broad array of 15 arthropod families and one nematode, with enrichment of glycoprotein genes in select insect lineages associated with retrotransposons that facilitated their capture and amplification [66], indicating that these viruses have had long-standing and ancient associations with invertebrates.) Our analysis identified six well-supported and independent lineages of *Chuviridae* EVEs within tick genomes: (1) a glycoprotein-encoding element with homology to Blacklegged tick chuvirus-2 (genus *Nigecruvirus*) in all three *Ixodes* species studied; (2) a lineage of elements with top hits to ORF3 (otherwise reported as a nucleoprotein, N gene) of the previous *Nigecruvirus* contained in the same *Ixodes* species; (3) a lineage comprising ORF3/hypothetical protein-encoding elements with top hits to members of genus *Mivirus* in all three *Ixodes* species, sister to a single Morsuvirus-like element detected in *I. persulcatus*; (4) a glycoprotein-encoding element from the three Ixodes species with homology to the latter *Mivirus* strains; (5) a nucleoprotein-encoding element with top hits to various Mivirus strains present in all three *Rhipicephalus* species examined + *D. silvarum* and *H. longicornis*; and (6) an RNA-dependent RNA polymerase-encoding element with top hits to various Mivirus strains detected in *H. longicornis* and *I. ricinus* (Figure 10A–F, respectively). This clade additionally contained three EVEs with top hits to a currently unclassified Liman tick virus (LMTV) that has been recovered within the *Chuviridae* in previous analyses [67] within *H. longicornis* and a Deer tick mononegavirales-like virus (DTMV) element in *I. persulcatus*.

### 4.6. Totiviridae (dsRNA)

**Figure 11:**
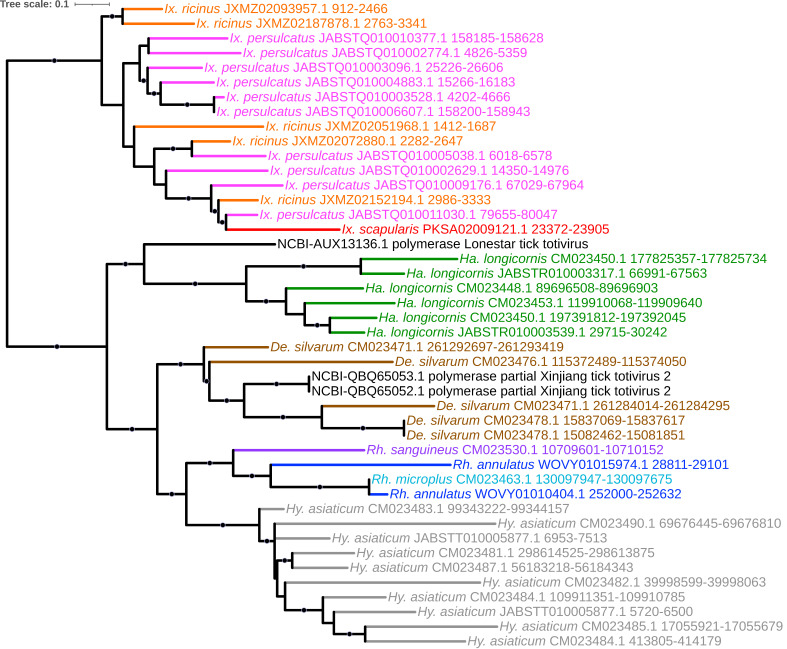
Sub-clade of phylogeny generated in Figure 2 illustrating shared ancestry of *Totiviridae* EVEs identified in tick genomes with varying homology to the RNA polymerase gene of both Xinjiang tick totivirus 2 and Lonestar tick totivirus in all five tick genera. Nodes with bootstrap supports ≥90 are represented with black circles. Taxon labels are formatted and color-coded by tick species as shown in Figure 3.

*Totiviridae* comprises double-stranded RNA viruses that are predominantly associated with fungi and protozoa but have more recently been discovered in insects and other arthropods [68]. Our analysis identified a clade of viral elements with varying homology to the RdRp gene of both Xinjiang tick totivirus 2 (isolated from ticks in Xinjiang, China) and Lonestar tick totivirus (isolated from *Am. americanum* in the United States [55]) in all five tick genera (Figure 11). This phylogenetic branching order is intriguing in that it mirrors the tick genera themselves ((((*Hyalomma* + *Rhipicephalus*), *Dermacentor*), *Haemaphysalis*), *Ixodes*) [69] and thus indicates that this EVE may represent an ancient endogenization event that predated the divergence of major tick genera postulated to have occurred ca. 60–70 million years ago [70].

### 4.7. Orthomyxoviridae (-ssRNA)

**Figure 12:**
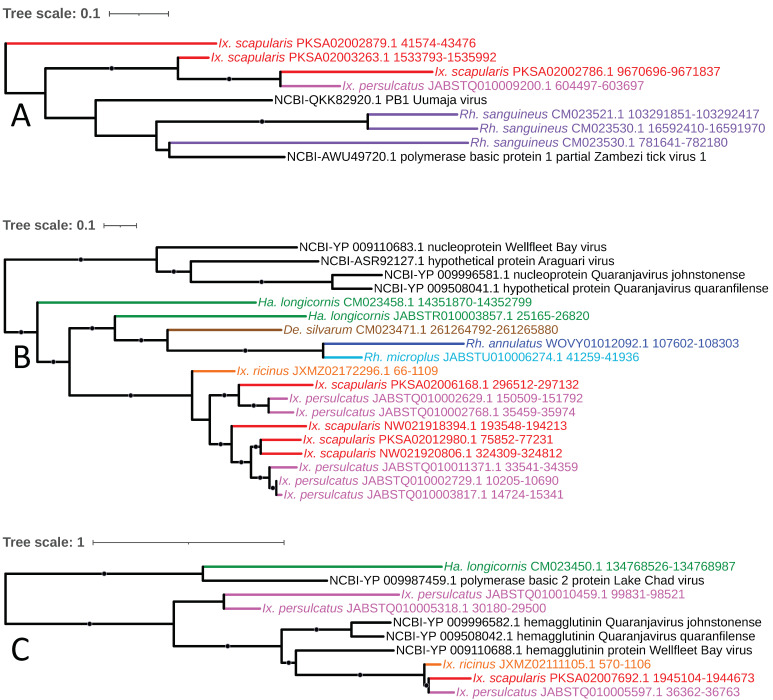
Sub-clades of phylogeny generated in Figure 2 illustrating shared ancestry of *Orthomyxoviridae* EVEs identified in tick genomes. These *Quaranjavirus*-like elements assort into a clade of polymerase basic protein 1 (PB1) homologs present in *Rh. sanguineus*, *Ix. persulcatus*, and *Ix. scapularis* with homology to Zambezi tick virus 1 and Uumaja virus (**A**). Nucleoprotein homologs in *Rh. microplus*, *Rh. annulatus*, *De. silvarum*, *Ha. longicornis*, and all three *Ixodes* species exhibit homology to Wellfleet Bay virus (WFBV; (**B**)). Hemagglutinin homologs were identified in the three *Ixodes* species with a single element in *Ha. longicornis* (**C**). Nodes with bootstrap supports ≥ 90 are represented with black circles. Taxon labels are formatted and color-coded by tick species as shown in Figure 3.

Three separate lineages of *Orthomyxoviridae* comprised predominantly of Quaranjavirus-like EVEs were identified: (1) a clade of polymerase basic protein 1 (PB1) homologs present in *R. sanguineus*, *I. persulcatus*, and *I. scapularis* with homology to Zambezi tick virus 1 (recently described from *Rhipicephalus* ticks in Mozambique [71]) and Uumaja virus; (2) two clades with homology to Wellfleet Bay virus (WFBV)—a clade of nucleoprotein homologs in *R. microplus*, *R. annulatus*, *D. silvarum*, *H. longicornis*, and all three *Ixodes* species; and (3) a clade of hemagglutinin homologs in the three *Ixodes* species with a single element in *H. longicornis* (Figure 12A–C, respectively). Wellfleet Bay virus is best known for its association with mass death of the common eider *Somateria mollissima* during the fall months in Cape Cod, Massachusetts, but can infect other birds as well and has been demonstrated to be capable of replicating in a variety of different animal cell lines (though no serious pathogenesis in any organism outside of eiders has been reported) [72,73]. It has been suggested that WFBV is vectored by either an argasid tick (a more conventional host for quaranjaviruses but with no species known to feed on seabirds in Massachusetts) or an ixodid tick (particularly *Ixodes uriae,* a well-documented ectoparasite of eider in Cape Cod) [73], especially given WFBV’s relationship with other quaranjaviruses, the presence of a baculoviral-derived gp64-like protein known to be uniquely adapted to arthropod transmission, and the recovery of quaranjaviruses in recent metatranscriptome analyses of hard ticks [74,75]. In addition to WFBV, a second bird-associated [76] quaranjavirus-like EVE was identified in our analysis, with homology to Johnston Atoll quaranjavirus in *H. longicornis* and *I. ricinus*.

Though the EVEs recovered here do not inherently demonstrate that hard ticks are vectors for any specific extant quaranjaviruses—indeed, the average amino acid similarity for all such hits was only 37.3%—their presence in these genomes does provide further evidence that similar viruses may be (or have been) encountered by ixodid ticks more frequently than their traditional association with the *Argasidae* suggests. If hard ticks do prove to be competent vectors for members of this genus, increased surveillance and study of these viruses may be useful in anticipating emerging disease in humans, livestock, and wildlife.

### 4.8. Flaviviruses (+ssRNA)

*Haemaphysalis longicornis* and *I. persulcatus* were both found to encode EVEs with homology to Alongshan virus; both VP1a and capsid protein-encoding elements were identified in *I. persulcatus*, while a single VP1a-encoding element was identified in *H. longicornis* (Supplementary Table S1; also reported by [77]). Human cases of Alongshan virus were first recorded in 2017 from Northeastern China among patients presenting with a variety of nonspecific febrile symptoms [78], and the pathogen has since been found in cattle and sheep in Northeastern China, where ~25% of the animals tested were positive for viral RNA [79]. It has also been detected in ticks from Russia [80], Finland [81], and France but with no accompanying human seropositivity in any of these areas (with the exception of a single French individual [82]). *I. persulcatus* and *I. ricinus* have been identified as potential vectors [78,81]. Thus, these elements are likely derived from past associations between these tick species, including *H. longicornis*, and relatives of human pathogenic viruses. Because Alongshan virus is an emerging human disease, further investigating the patterns of EVEs in various tick populations may provide additional information on how this and other jingmenviruses are maintained by these vector arthropods in the environment and whether Alongshan or Alongshan-like sequences are being encountered and/or integrated in current lineages (or are strictly relics of past encounters with similar viruses). Two additional elements with homology to the RdRP gene of Mogiana tick virus (a lineage of Flavivirus known as jingmenviruses [82]) were identified in *I. ricinus*; the two elements encode separate regions of the N- and C-terminal regions of the RdRP. Closer inspection revealed that these two HSPs span the entirety of two small scaffolds in the *I. ricinus* assembly used here (JXMZ02010324.1; 1629 bp and JXMZ02142294.1; 2602 bp) and maintain an AA similarity of 98.0–99.6% to Mogiana tick virus, suggesting that this element is either another example of a very recent endogenization event or, alternatively, that these *I. ricinus* scaffolds are themselves viral in origin as opposed to genomic. Although this EVE was detected in the *I. ricinus* genome, it was not found in the tick from which the virus was originally isolated and reported (*R. microplus* [83]), further highlighting the fact that additional variables specific to different ticks and their environments may influence both the opportunity for viral encounters and the probability of incorporation into the genomes of somatic cells and/or the germline.

### 4.9. Other/Unassigned Viruses

**Figure 13:**
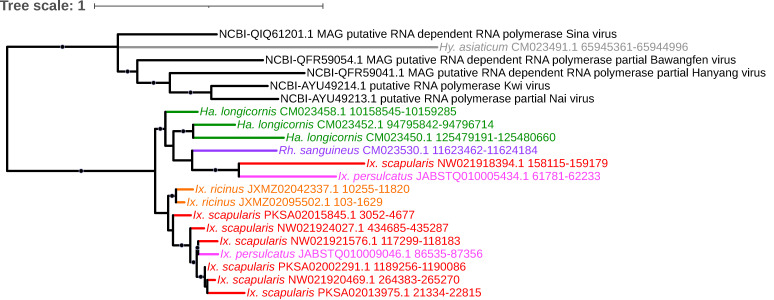
Sub-clade of the phylogeny generated in Figure 2 illustrating shared ancestry of EVEs identified in tick genomes composed entirely of RNA polymerase elements with homology to multiple members of a novel segmented +ssRNA viral lineage provisionally described as family *Quenyaviridae* and present in all three *Ixodes species*, *R. sanguineus*, and *H. longicornis*. Nodes with bootstrap supports ≥90 are represented with black circles. Taxon labels are formatted and color-coded by tick species as shown in Figure 3.

Many of the EVEs identified in this analysis corresponded to viral lineages that have been poorly described and/or solely documented through either virome sequencing of different tick species or analysis of various metagenomic datasets. One such example is a clade composed entirely of RdRP elements with homology to multiple members of a novel segmented +ssRNA viral lineage recently described by [84] and provisionally referred to as Quenyaviruses (family *Quenyaviridae*). These elements were present in all three *Ixodes* species, *R. sanguineus*, and *H. longicornis* (Figure 13). Three of the reference viral hits (Kwi virus, Nai virus, Sina virus, and Hanyang virus) were isolated from arthropods, while Bawangfen virus was described from a lizard dataset [84]. Whether these elements collectively represent integrations by multiple individual Quenyavirus or one or more closely related yet undescribed viruses having undergone post-endogenization sequence degradation remains unclear.

**Figure 14:**
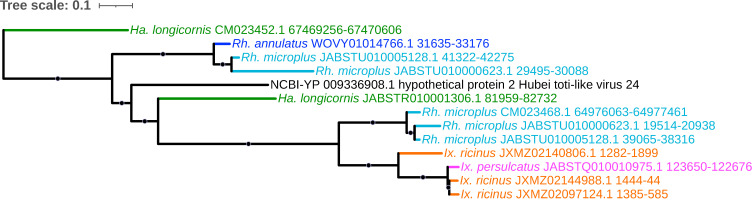
Sub-clade of phylogeny generated in Figure 2 illustrating shared ancestry of toti-like virus EVEs identified in tick genomes, showing multiple elements present within the *I. ricinus*, *I. persulcatus*, *R. annulatus*, *R. microplus*, and *H. longicornis* genomes with homology to hypothetical protein 2 of Hubei toti-like virus 24. Nodes with bootstrap supports ≥90 are represented with black circles. Taxon labels are formatted and color-coded by tick species as shown in Figure 3.

Another such currently unclassified viral lineage identified here, comprising multiple EVE elements in four tick species, is that of the toti-like viruses (Figure 14); twelve total elements that are present within the *I. ricinus*, *I. persulcatus*, *R. microplus*, and *H. longicornis* genomes were identified with homology to hypothetical protein 2 of Hubei toti-like virus 24, isolated from a pool of mixed tick species by [85]. *Totiviridae* is a family consisting of dsRNA viruses that are predominantly associated with fungi and protozoa but have been recently discovered in insects and other arthropods [86]. No such toti-like virus-derived EVEs were identified in *I. scapularis*, as was also reported in the analysis of Russo et al. [62].

## 5. Conclusions

The preceding analysis explores a rich dataset of EVE sequences from which many paleovirological phenomena can be inferred and offers valuable reference information for future -omics analyses of ticks that may misidentify EVEs as genetic information from actively replicating viruses; however, further exploration is needed to infer whether any of the elements identified are directly relevant to vector immunology. Even if the utility of arthropod EVEs in regulating viral infection has been demonstrated [16], the putative EVEs identified in this analysis may or may not retain (or have ever possessed) a functional role in their respective ticks. In order to have any immunological utility, a transcribed EVE presumably needs to maintain a certain degree of homology to the target virus. PIWI-interacting RNA epigenetic regulation of chromatin in *Drosophila melanogaster* can tolerate a limited number of mismatches, but its ability to bind a target sequence still rapidly depreciates as they accumulate across the strand [87]. It has yet to be determined whether the same degree of sequence specificity is required for antiviral function in other arthropods. Small interfering RNA (siRNA) activity (which has also been implicated in arthropod viral control [88]) might be a closer functional analog and also demonstrates some tolerance to mismatches depending on their exact position in the RNA; other forms of RNAi with less sequence specificity can similarly inhibit translation even if they cannot effectively guide cleavage proteins [89,90]. Regardless of how this may or may not be reflected in piRNA mechanisms, homology with published extant viral sequences remains so low for the vast majority of tick EVE loci identified that present-day involvement with the PIWI-interacting RNA pathway against the NCBI reference viruses with which they were identified would be highly unlikely, thus reinforcing the idea that most EVEs identified in this way represent relics of ancestral infections inherited via the germline rather than an active antiviral repertoire. However, this also re-emphasizes an important caveat regarding certain paleovirological inferences: in cases of low-to-moderate sequence homology to extant viruses, the strains associated are unlikely to be the source of the original viral integration and may possess different properties in terms of host range and functionality.

It is also possible that the overall body of putative EVEs identified here represents only a snapshot of the potential endogenous viral elements an individual (or even a single cell within an individual) could possess at any given time, shaped both by ancestral germline incorporation and the viruses encountered within various tissues over the course of an individual tick’s lifetime. Studies in mosquitoes have revealed that EVE composition in wild-caught samples may vary based on both phylogeographic lineage and encounters with contemporary viruses in their environment [91]; future research should explore how this principle may apply to tick EVEs in samples from diverse geographic locations. Utility of individual EVEs in these ticks could also be confirmed through small RNA sequencing (as has also been accomplished in mosquitoes [16]), and a further analysis of flanking genomic DNA associated with the EVEs presented here could assess the extent to which they are associated with mobile elements such as active transposons capable of facilitating their incorporation.

As complete genomes have been generated from only a fraction of the many tick species implicated in the spread of human and livestock diseases, future studies will continue to explore how EVE integration varies geographically and temporally within and among tick species and how this information may be effectively utilized to enhance our capacity for vector control via emerging technologies such as RNA-mediated silencing.

## Figures and Tables

**Table 1:** Genomes screened for the presence of endogenous viral elements. Accession numbers prefixed with GCF indicate assemblies retrieved from RefSeq, while GCA indicates a GenBank assembly. Numbers marked with an asterisk represent contigs and not scaffolds.

Tick Species	NCBI Accession	Chromosomal Scaffolds	Unplaced Scaffolds	Unique EVE Loci *(Adintoviridae)*	Reference
*Dermacentor silvarum*	GCF 013339745.1	11	1653	33 (0)	[19]
	GCA 013339745.1				
*Haemaphysalis longicornis*	GCA 013339765.1	11	3878	56 (1)	[19]
*Hyalomma asiaticum*	GCA 013339685.1	11	6313	39 (0)	[19]
*Ixodes persulcatus*	GCA 013358835.1	0	11,601	400 (221)	[19]
*Ixodes ricinus*	GCA 000973045.2	0	204,516	121 (36)	[27]
*Ixodes scapularis*	GCF 002892825.2	0	13,270 *	381 (195)	[28]
	GCA 002892825.2		6476 *		
*Rhipicephalus annulatus*	GCA 013436015.1	0	16,339 *	98 (0)	[29]
*Rhipicephalus microplus*	GCF 013339725.1	11	7036	48 (0)	[19]
	GCA 013339725.1				
*Rhipicephalus sanguineus*	GCA 013339695.1	11	2317	50 (0)	[19]

## Data Availability

The NCBI accession numbers for the tick genomes screened in this work are available in Table 1. The raw blast output, amino acid sequence alignment, and resultant phylogenetic tree are available from the Mendeley Data repository, V1, doi:10.17632/vdh9bgdb6h.1.

## Supplementary Materials

The following supporting information can be downloaded at https://www.mdpi.com/article/10.3390/v15112201/s1, Table S1: DIAMOND (BLASTX algorithm) homology search output for all identified endogenous viral elements in this study after the addition of 200 bp flanking sequence on each end of the initial TBLASTN HSP. Data in columns T–AJ would indicate that the element had a top-hit to a sequenced ORF in a tick or eukaryote genome (indicated in column B). The top viral hit data for each query are contained in columns C–S; Table S2: Provenance and distribution of endogenous viral elements in hard tick genomes. Numbers indicate the number of unique EVEs identified in each species, with total counts per species summarized below; Figure S1: EVE phylogeny (as shown in Figure 2) with full tip labels that include NCBI taxonomic string and annotation, manually rooted on the *Polydnaviridae*. Nodes with solid circles indicate bootstrap support ≥90; Figure S2: Maximum-likelihood phylogeny of adintoviral elements identified in this study. Nodes with bootstrap supports ≥90 are represented with black circles. Tip labels for EVEs are of the format “(tick species) (scaffold accession) (coordinates)”; labels for the NCBI top hits are of the format “NCBI—(accession) (annotation) (virus)”. The tree is manually rooted on an outgroup clade of Parvoviridae (collapsed); Figure S3: Maximum-likelihood phylogeny of iridoviral elements identified in this study. Tip labels are as shown in Figure S2.
