# Supplementary material for: Endogenous Viral Elements in Ixodid Tick Genomes
Source: Viruses. 2023 Oct 31;15(11):2201. doi: 10.3390/v15112201 (PMC10675110; doi:10.3390/v15112201)
Supplement: Supplementary file 1 [file viruses-15-02201-s001.zip › FIGURE S1.pdf]

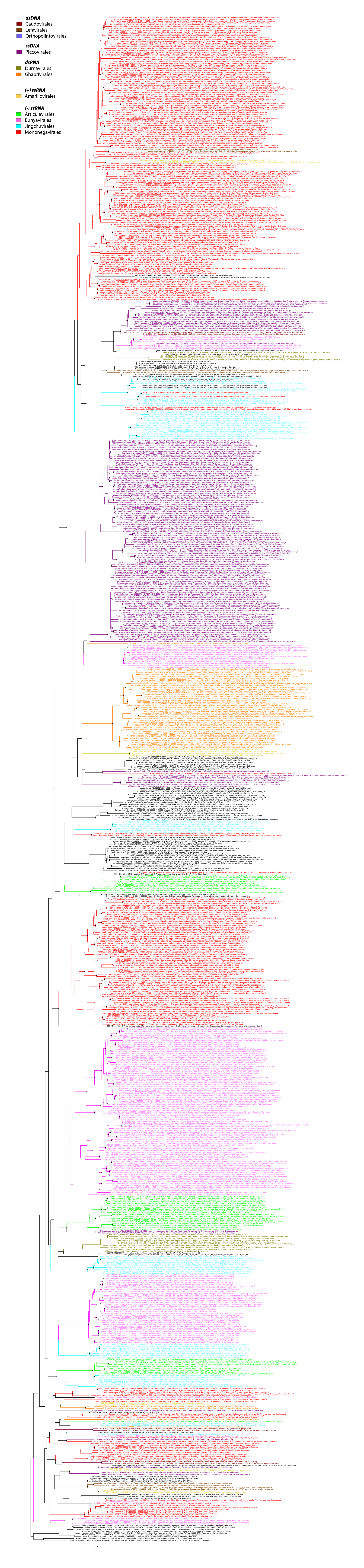
dsDNA
Caudovirales
Lefavirales
Orthopolintovirales
ssDNA
Piccovirales
dsRNA
Durnavirales
Ghabrivirales
(+) ssRNA
Amarillovirales
(-) ssRNA
Articulavirales
Bunyavirales
Jingchuvirales
Mononegavirales
