# Supplementary figures and images for: Endogenous Viral Elements in Ixodid Tick Genomes
Source: Viruses. 2023 Oct 31;15(11):2201. doi: 10.3390/v15112201 (PMC10675110; doi:10.3390/v15112201)

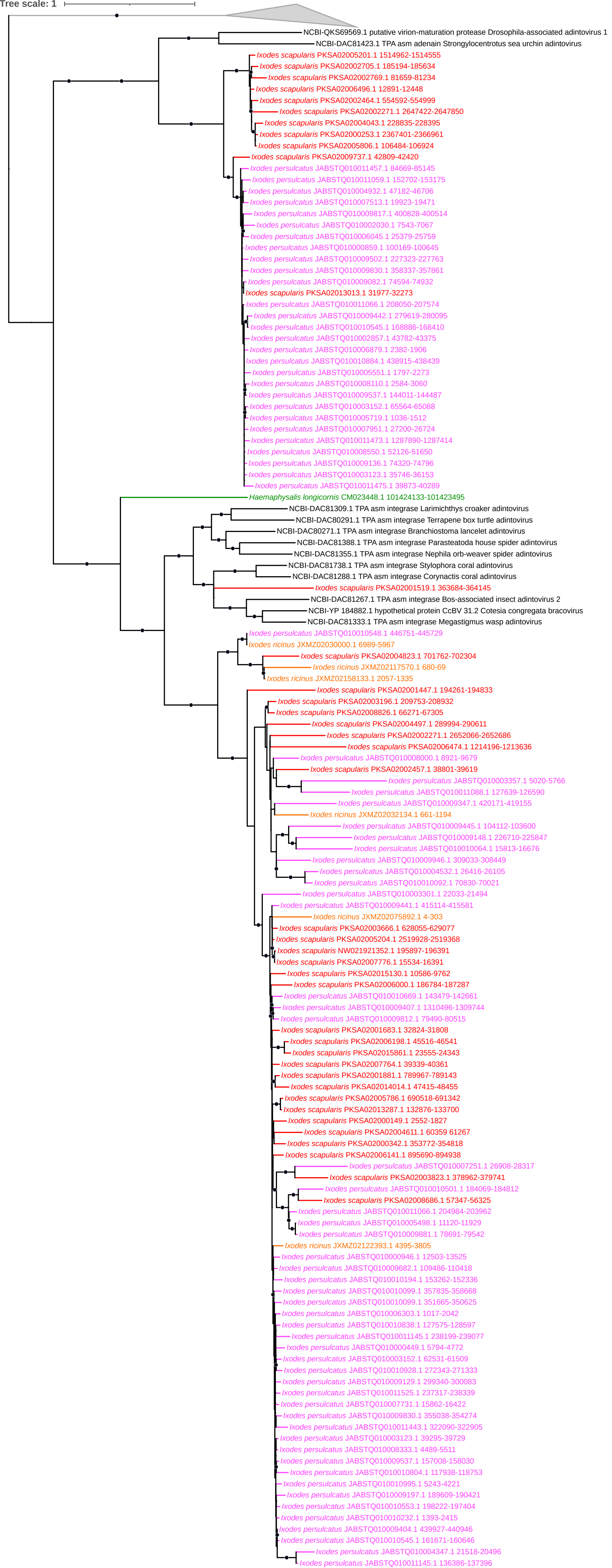

Supplement: Supplementary file 1 [file viruses-15-02201-s001.zip › FIGURE S2.pdf]

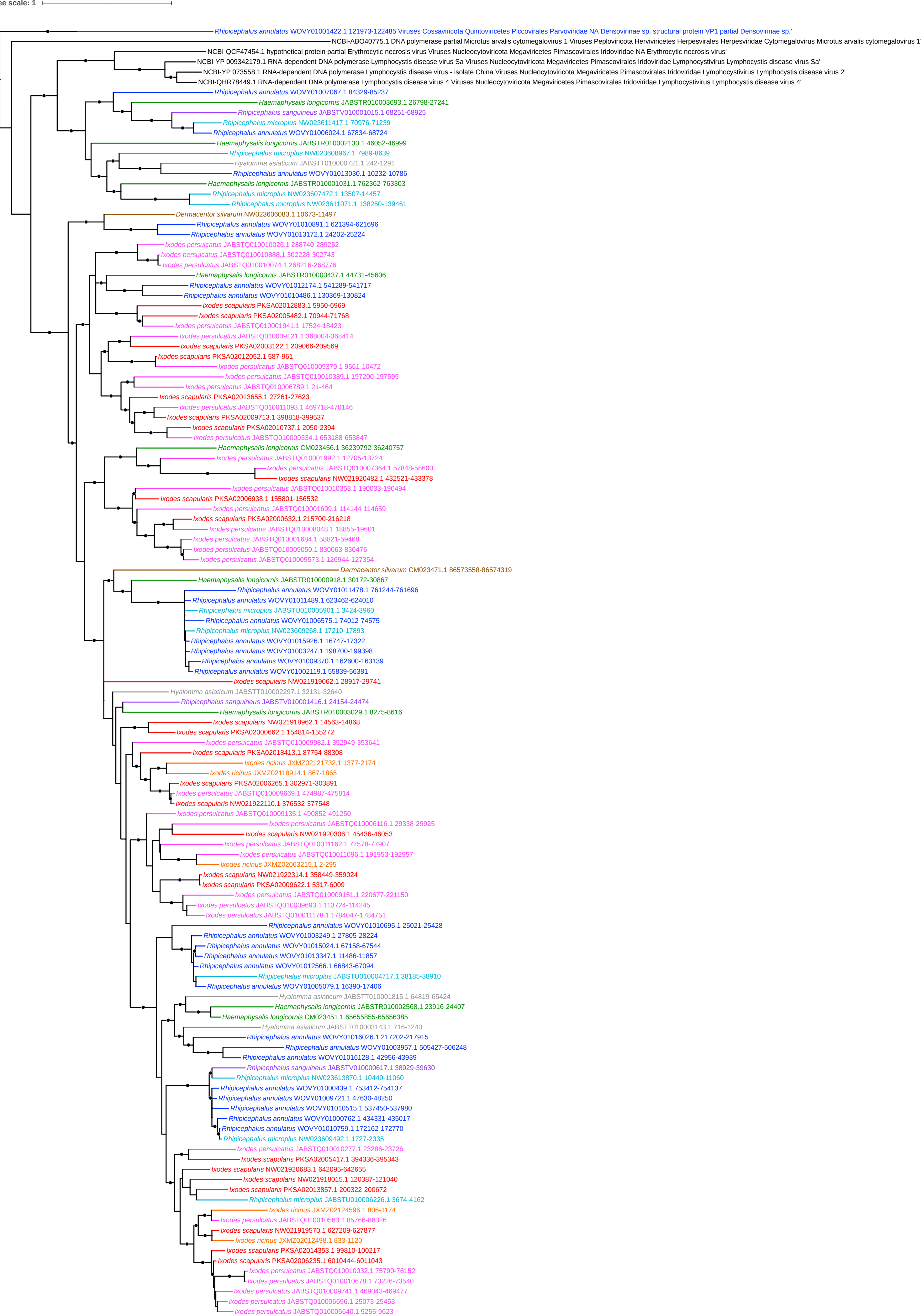

Supplement: Supplementary file 1 [file viruses-15-02201-s001.zip › FIGURE S3.pdf]
